# Traditional Chinese herbal formulas modulate gut microbiome and improve insomnia in patients with distinct syndrome types: insights from an interventional clinical study

**DOI:** 10.3389/fcimb.2024.1395267

**Published:** 2024-05-16

**Authors:** Huimei Zeng, Jia Xu, Liming Zheng, Zhi Zhan, Zenan Fang, Yunxi Li, Chunyi Zhao, Rong Xiao, Zhuanfang Zheng, Yan Li, Lingling Yang

**Affiliations:** ^1^ Guangdong Provincial Hospital of Chinese Medicine, Guangdong Provincial Academy of Chinese Medical Sciences, and The Second Clinical College, Guangzhou University of Chinese Medicine, Guangzhou, China; ^2^ Singapore Institute for Clinical Sciences, Agency for Science, Technology and Research, Singapore, Singapore; ^3^ The Second Clinical Medical College, Guangzhou University of Traditional Chinese Medicine, Guangzhou, China; ^4^ Department of Rehabilitation, The Eighth People’s Hospital of Hefei, Hefei, China; ^5^ Teaching and research Center, Guangdong Provincial Trade Union Cadre School, Guangzhou, China

**Keywords:** insomnia, traditional Chinese medicine syndrome, herbal formula, gut microbiome, gut-brain axis, longitudinal integrative network

## Abstract

**Background:**

Traditional Chinese medicine (TCM) comprising herbal formulas has been used for millennia to treat various diseases, such as insomnia, based on distinct syndrome types. Although TCM has been proposed to be effective in insomnia through gut microbiota modulation in animal models, human studies remain limited. Therefore, this study employs machine learning and integrative network techniques to elucidate the role of the gut microbiome in the efficacies of two TCM formulas — center-supplementing and qi-boosting decoction (CSQBD) and spleen-tonifying and yin heat-clearing decoction (STYHCD) — in treating insomnia patients diagnosed with spleen qi deficiency and spleen qi deficiency with stomach heat.

**Methods:**

Sixty-three insomnia patients with these two specific TCM syndromes were enrolled and treated with CSQBD or STYHCD for 4 weeks. Sleep quality was assessed using the Pittsburgh Sleep Quality Index (PSQI) and Insomnia Severity Index (ISI) every 2 weeks. In addition, variations in gut microbiota were evaluated through 16S rRNA gene sequencing. Stress and inflammatory markers were measured pre- and post-treatment.

**Results:**

At baseline, patients exhibiting only spleen qi deficiency showed slightly lesser severe insomnia, lower IFN-α levels, and higher cortisol levels than those with spleen qi deficiency with stomach heat. Both TCM syndromes displayed distinct gut microbiome profiles despite baseline adjustment of PSQI, ISI, and IFN-α scores. The nested stratified 10-fold cross-validated random forest classifier showed that patients with spleen qi deficiency had a higher abundance of *Bifidobacterium longum* than those with spleen qi deficiency with stomach heat, negatively associated with plasma IFN-α concentration. Both CSQBD and STYHCD treatments significantly improved sleep quality within 2 weeks, which lasted throughout the study. Moreover, the gut microbiome and inflammatory markers were significantly altered post-treatment. The longitudinal integrative network analysis revealed interconnections between sleep quality, gut microbes, such as *Phascolarctobacterium* and Ruminococcaceae, and inflammatory markers.

**Conclusion:**

This study reveals distinct microbiome profiles associated with different TCM syndrome types and underscores the link between the gut microbiome and efficacies of Chinese herbal formulas in improving insomnia. These findings deepen our understanding of the gut-brain axis in relation to insomnia and pave the way for precision treatment approaches leveraging TCM herbal remedies.

## 1 Introduction

Insomnia, a condition marked by dissatisfaction with sleep duration, continuity, and quality, is characterized by persistent difficulties in falling asleep or maintaining sleep, coupled with daytime functional impairment (Perlis et al., 2022). As the most common sleep disorder, insomnia is highly prevalent, affecting approximately 30%–50% of the general population (Brownlow et al., 2020). Often present independently or co-occurring with other medical conditions, such as cardiometabolic diseases, or mental health disorders, such as depression or anxiety, insomnia poses a significant risk of the development and exacerbation of these conditions if left untreated (Perlis et al., 2022).

The first-line recommended treatment for insomnia is cognitive behavioral therapy for insomnia, but access to this therapy is often limited due to high costs and variable response rates (Wilson et al., 2010). As a second-line treatment, pharmacotherapy, particularly hypnotics, is frequently prescribed (Madari et al., 2021; Sutton, 2021; Perlis et al., 2022). Despite their relative safety for long-term use, the long-term adverse effects and varying efficacy of hypnotic medications remain a concern (Yue et al., 2023). There is no global consensus on the most effective pharmacological treatment with the best risk-benefit ratio (Perlis et al., 2022). This complexity underscores the necessity to explore different nonpharmacologic and pharmacologic treatments, especially with the emergence of more effective interventions (Yue et al., 2023).

Current understanding of the neurobiological mechanisms underlying insomnia is still evolving. The central system, which controls the sleep-wake cycle, is influenced by signals from peripheral tissues. Recent research has revealed reciprocal connections between the central nervous system, sleep, and the immune system. This relationship implies that while sleep bolsters immune defenses, afferent signals from immune cells also promote sleep. The homeostatic regulation of sleep is influenced by cytokine responses, neuroendocrine and autonomic pathways, and inflammatory peptides, collectively forging a link between sleep and the immune system (Irwin, 2019; Garbarino et al., 2021). Additionally, emerging studies suggest that the microbiota-gut-brain axis plays a regulatory role in sleep behavior, highlighting its potential significance in understanding sleep disorders (Wang et al., 2022). Notably, sleep deprivation can negatively affect gut microbiome function, and alterations in gut microbiota have been observed in sleep disorders (Feng et al., 2023).

Traditional Chinese medicine (TCM) has been used to treat insomnia for over 2000 years, and it continues to gain attention in modern medical practices (Liu et al., 2017). Historical medical books and recent studies have confirmed the efficacy of various TCM formulas and herbs in enhancing sleep (Singh and Zhao, 2017b). TCM treatments are customized based on individual pattern diagnosis or syndrome differentiation, which involves analyzing an individual’s symptoms, signs, pulse form, and tongue appearance. Given the diversity of symptoms and signs, multiple TCM pattern diagnoses can exist for the same disease, leading to varied treatment approaches (World Health Organization, 2007). There are different TCM prescriptions for different TCM syndrome types for insomnia (Yeung et al., 2012). Spleen qi deficiency syndrome, including spleen qi deficiency and spleen qi deficiency with heat stagnation, is a prevalent TCM syndrome type in insomnia cases (Singh and Zhao, 2017a). Previous studies have highlighted a significant correlation between spleen inadequacy and imbalances in gut microbiota (Qiu et al., 2017; Lin et al., 2018). Recent evidence in animal models suggests that TCM can improve sleep quality by regulating gut microbiota (Si et al., 2022a, Si et al., 2022b).

The center-supplementing and qi-boosting decoction (CSQBD) and spleen-tonifying and yin heat-clearing decoction (STYHCD) are two classic TCM formulas recorded in the “Treatise on Spleen and Stomach” by Li Gao of the Jin dynasty (1115–1234). They have been traditionally used to address the imbalances of qi and yin that are often observed in sleep disorders according to TCM principles. Specifically, CSQBD is used to treat spleen qi deficiency, whereas STYHCD addresses spleen qi deficiency with heat stagnation. However, a critical research gap persists, especially in human studies, regarding the association of different spleen qi deficiency syndrome types with distinct gut microbiome profiles. Moreover, the gut microbiome-modulating efficacy of various herbal formulas to treat different TCM syndrome types in insomnia remains largely unexplored.

In addressing the identified research gap, this study endeavors to elucidate the relationship between different TCM syndrome types and their specific gut microbiome profiles in the context of insomnia through a clinical trial. Additionally, it aims to evaluate the role of the gut microbiome in the treatment of insomnia among patients classified by specific TCM syndromes, utilizing two targeted herbal formulas CSQBD and STYHCD. These investigations aim to deepen our understanding of the interplay between the therapeutic efficacy of TCM for insomnia and the microbiota-gut-brain axis, which could provide novel insights to refine the precision of therapeutic interventions for insomnia.

## 2 Materials and methods

### 2.1 Study design

This is a two-arm interventional trial involved 63 patients with insomnia. Patients were recruited and divided into two groups based on their TCM syndromes: 28 patients with spleen qi deficiency-associated insomnia received the CSQBD and 35 patients with spleen qi deficiency and stomach heat-associated insomnia were treated with the STYHCD. This study adhered to the principles of the declaration of Helsinki and received approval from the ethics committee of Guangdong Provincial Hospital of Chinese Medicine (ChiCTR-INR-1701110).

Exclusion criteria for the study included individuals with *Diabetes mellitus*, hypertension, cardiovascular diseases (based on clinical history), those on sleep medications, or those who had used antibiotics in the 6 months preceding the study. Additionally, patients whose insomnia was attributed to mental disorders, physical disorders, or medication use were also excluded from the analysis.

The herbal formulas of STYHCD and CSQBD are demonstrated in **Supplementary Tables S1** and **S2**. They were processed into decocting-free granules according to a standard production process (Supplementary Materials) and administered orally with hot water — two bags/dose, twice a day. Both formulas were supervised by Guangdong Provincial Hospital of Chinese Medicine and produced by Jiangyin Tianjiang Pharmaceutical Co., Ltd., ensuring quality control. The major chemical components of these formulas were identified using high performance liquid chromatography-mass spectrometry, with details provided in the **Supplementary Methods** and **Supplementary Figures S1** and **S2**.

Fecal samples (>500 mg each) were collected at 0, 2, and 4 weeks post-interventions using microlution (Dayun Gene Technology, Shenzhen, China) stool collection tubes containing stool DNA stabilizer. All samples were processed within the temperature range and timeframe suggested by the manufacturer’s instruction. Samples were stored at −80°C for subsequent gut microbiome analysis. Plasma samples were collected at the baseline (week 0) and at the end (week 4) of the study to assess stress, inflammatory, and anti-inflammatory makers by ELISA.

### 2.2 Sleep quality assessment

To evaluate the sleep quality of patients, we employed two well-established measurement methods: the Pittsburgh Sleep Quality Index (PSQI) and Insomnia Severity Index (ISI). The total global PSQI score, which ranges from 0 to 21, is used to quantify sleep quality, with a score >7 indicating poor sleep quality (Buysse et al., 1989). It is a comprehensive assessment that measures seven dimensions of sleep: subjective sleep quality (good or poor), sleep latency (≤15 to >60 min), sleep duration (≥7 to <5 h), sleep efficiency (≥85% to <65% h sleep/h in bed), sleep disturbances (any kind of sleep disturbance ≥1 time/week), and use of sleeping medications (use of sleep medication ≥1 time/week). The ISI was used to assess the severity of both nighttime and daytime insomnia (Morin et al., 2011). The efficacy of the herbal formulas in treating insomnia was evaluated by comparing these scores before 2 and after 4 weeks of the treatment.

### 2.3 Fecal DNA extraction and 16S rRNA gene sequencing

The genomic DNA samples of the gut microbiota were extracted using the DNeasy PowerSoil Kit (QIAGEN Inc., Netherlands). The amplification of the V3-V4 region of the 16S rRNA gene was carried out using the 341F forward primer (5’-CCTACGGGNGGCWGCAG-3’) and the 806R reverse primer (5’-GGACTACHVGGGTATCTAAT-3’) with minor modifications (Tong et al., 2018). The purification of PCR amplicons was carried out using Agencourt AMPure beads (Beckman Coulter, Indianapolis, IN), and the quantification of the PCR amplicons was performed using the PicoGreen dsDNA assay kit (Invitrogen, Carlsbad, CA, USA). Subsequently, the quantified amplicons were pooled in equal amounts. Paired-end sequencing of 2×250 bp was conducted using the Illumina MiSeq platform and the MiSeq reagent kit v3 (Illumina, San Diego, CA, USA) at Shanghai Personal Biotechnology Co., Ltd.

### 2.4 Microbiome data processing and bioinformatics

Most of enrolled patients provided fecal samples at all the three time points, resulting in a total of 136 fecal samples for gut microbiome analysis. Among these, the CSQBD group contributed 26, 26, and 22 samples at 0, 2, and 4 weeks, respectively, whereas the STYHCD group contributed 21, 20, and 21 samples at 0, 2, and 4 weeks, respectively. The initial raw sequencing data was processed using QIIME2 (v2023.2) (Bolyen et al., 2019; Chen et al., 2020; Xu et al., 2020). The amplicon sequence variants (ASV) were obtained with the DADA2 plugin (Friedman and Alm, 2012). The taxonomic classification of all ASV representative sequences was performed using a Naive Bayes classifier trained on the V3-V4 region of the 16S rRNA gene with the SILVA database v138.1 (Quast et al., 2013; Robeson et al., 2021). The phylogenetic tree was constructed using the SEPP method within the fragment-insertion plugin (Matsen et al., 2010; Eddy, 2011; Matsen et al., 2012; Janssen et al., 2018). Following rigorous data processing and quality control procedures, 6,540,042 high-quality reads were retained, averaging 48,089 ± 7,669 reads/sample. A total of 891 features were subsequently utilized for downstream analysis. To mitigate discrepancies in varying sequencing depths among the samples, the ASV abundance table was rarefied to the same sequencing depth of 33,000 for downstream analysis. The diversity plugin in QIIME2 was used for the generation of alpha-diversity indices, beta-diversity distance matrices, and ordination matrices through the core-metrics-phylogenetic method. The differential gut microbiome resulting from both herbal formulas over time was identified by using random forest regressor with q2-sample-classifier (Bokulich et al., 2018) — a nested stratified 10-fold cross-validation approach with 500 decision trees. The seed used by random number generator was 123.

### 2.5 Statistical analysis

The demographics and baseline characteristics between the two treatment groups were compared using the Mann-Whitney U test for continuous variables and chi-square test for categorical variables. A linear mixed model was applied to assess the longitudinal changes in PSQI, ISI, inflammatory markers, differential microbes, and alpha-diversity indices in both treatment groups. The subject ID was included as a random effect, whereas time was considered as a fixed effect. This analysis was conducted in R (version 4.3.0) using the lmerTest package (Kuznetsova et al., 2017). To compare the longitudinal effects of the two herbal formulas, the same methodology was applied. The interaction between time and treatment was included as the fixed effect to investigate potential differences in treatment responses. Prior to analysis, all data underwent log10 transformation. To explore the longitudinal association between PSQI, ISI, stress or inflammatory markers, and microbes, we used the rmcorr package in R (version 4.3.0) (Bakdash and Marusich, 2017). The association between alpha-diversity indices/microbial species and drug treatments was recognized with MaAsLin2 (Mallick et al., 2021). The Adonis test was performed with the vegan package in R. Additionally, to address multiple comparisons, the Benjamini-Hochberg method was used to correct p-values. To visualize associated networks, Cytoscape v3.9.1 was used, constructing an informative representation of interrelationships revealed by the data.

## 3 Results

### 3.1 Insomnia patients with different TCM syndromes harbored different gut microbiome profiles

**Figure 1:**
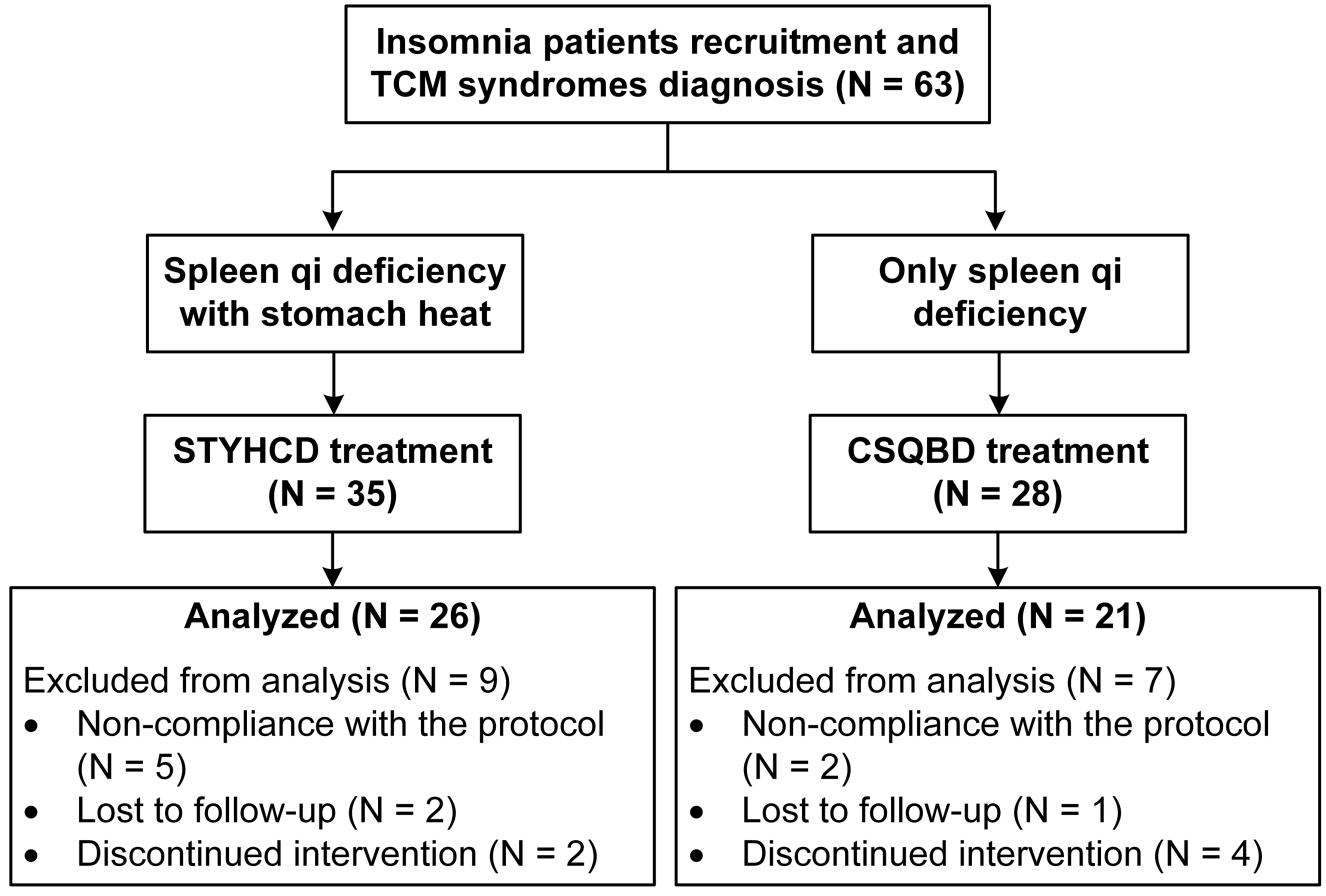
Clinical interventional trial flow diagram. STYHCD, spleen-tonifying and yin heat-clearing decoction; CSQBD, center-supplementing and qi-boosting decoction.

Of the 63 insomnia patients, 47 patients completed the 4-week treatment period and were included in the data analysis (**Figure 1**). Demographic and baseline characteristics indicated slightly more severe insomnia in patients with spleen qi deficiency and stomach heat than in patients with only spleen qi deficiency, as indicated by PSQI and ISI scores. However, this difference became statistically insignificant after adjusting for multiple comparisons (**Table 1**). Patients with spleen qi deficiency and stomach heat syndrome exhibited significantly higher INF-α levels at the baseline than patients with only spleen qi deficiency. Rest of the demographic and baseline characteristics were comparable between the two treatment groups at the baseline.

**Table 1:** Study demographics and baseline characteristics.

Variable		STYHCD (N = 26)	CSQBD (N = 21)	p-value^α^	FDR_BH^β^
Age (year)		36.0 ± 11.3	41.7 ± 11.6	0.1768	0.3789
Gender (N)	Female	18	15	1	1
	Male	8	6		
Education (N)	Below university	12	10	1	1
	University	14	11		
Systolic blood pressure (mmHg)		114.3 ± 13.7	114.5 ± 11.5	0.9896	1
Diastolic blood pressure (mmHg)		70.4 ± 8.2	70.9 ± 7.2	0.6945	0.9470
Heart rate (BPM)		71.9 ± 12.6	76.7 ± 9.7	0.1358	0.3563
PSQI score		13.6 ± 2.4	12.4 ± 2.6	0.0884	0.3315
ISI score		20.6 ± 4.0	17.6 ± 4.8	0.0328	0.2420
Cortisol (ng/mL)		19.3 ± 12.2	29.0 ± 19.7	0.0484	0.2420
IL-1β (pg/mL)		158.3 ± 66.6	185.4 ± 205.9	0.1425	0.3563
IL-6 (pg/mL)		57.2 ± 56.5	50.8 ± 17.4	0.2567	0.4345
TNF-α (pg/mL)		56.7 ± 29.7	65.4 ± 78.9	0.2607	0.4345
TNF-β (pg/mL)		26.5 ± 44.1	41.5 ± 67.3	0.9429	1
IFN-α (pg/mL)		93.0 ± 87.4	36.4 ± 50.2	0.0003	0.0045
IL-10 (pg/mL)		22.1 ± 19.9	14.3 ± 6.8	0.3748	0.5622

The table displayed the number of subjects for each categorical variable — gender and education. The rest of the variables were shown as the mean ± standard deviation.

STYHCD, spleen-tonifying and yin heat-clearing decoction; CSQBD, center-supplementing and qi-boosting decoction.

^α^ p-values were obtained with Mann-Whitney U test for continuous variables and chi-square test for categorical variables.

^β^FDR_BH, FDR-corrected p-values were obtained using Benjamini-Hochberg method for adjusting multiple comparisons.

**Figure 2:**
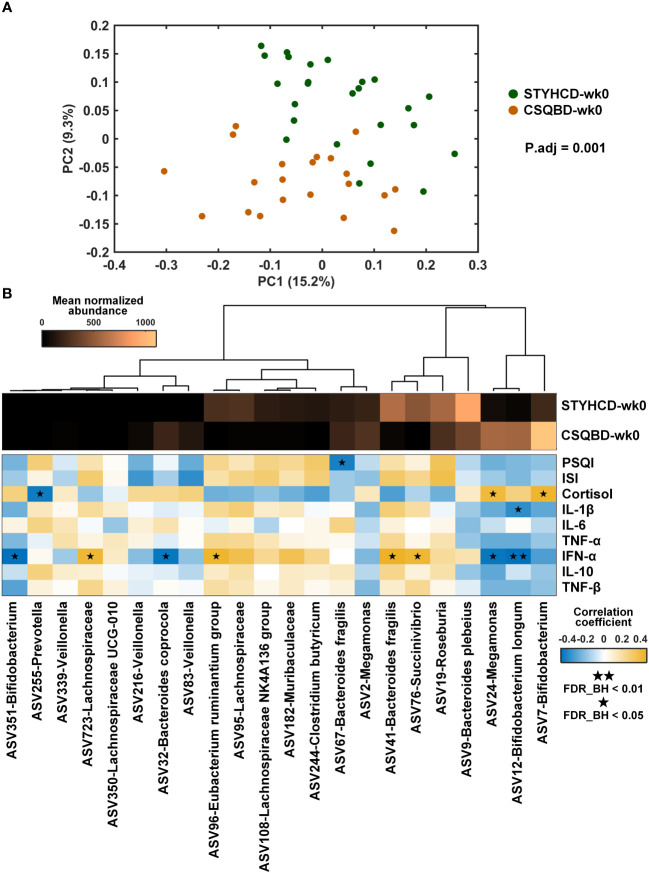
Baseline gut microbial profiles in insomnia patients with different TCM syndromes. **(A)** Principal coordinate analysis based on unweighted UniFrac distance. Adjusted P-value (P.adj) was obtained after controlling the baseline scores of PSQI, ISI, and IFN-α using the Adonis test. **(B)** Heatmap of the top 22 differential gut microbial species between the two treatment groups at the baseline and their correlations with clinical measurements. The abundance of each ASV was averaged within each group. Correlation coefficient was obtained with spearman correlation. FDR_BH, FDR-corrected p-value obtained using the Benjamini-Hochberg method to adjust for multiple comparisons. STYHCD, spleen-tonifying and yin heat-clearing decoction; CSQBD, center-supplementing and qi-boosting decoction; ASV, amplicon sequence variant.

Regardless of minor differences in the insomnia levels, striking differences in gut microbiome profiles of patients with the two TCM syndromes (P.adj = 0.001, **Figure 2A**) were observed. This finding was based on the Unweighted UniFrac distance and adjusted for baseline differences in PSQI, ISI, and IFN-α scores using the Adonis test. Besides, patients with only spleen qi deficiency exhibited greater microbial diversity and evenness than patients with spleen qi deficiency and stomach heat (**Supplementary Table S3**). These findings suggest a correlation between specific TCM syndrome types in insomnia and gut microbiota profiles.

By using random forest classifier, we identified 22 differential microbial species in insomnia patients with different TCM syndromes (**Figure 2B**). The classification accuracy was 97.8%. Of these 22 ASVs, 11 species, including *Bifidobacterium longum*, *Bacteroides coprocola*, 2 ASVs of *Bifidobacterium*, 3 ASVs of *Veillonella*, and 1 ASV of *Prevotella*, were more abundant in the patients with only spleen qi deficiency than in patients with spleen qi deficiency and stomach heat. Conversely, 11 variants, including 4 ASVs from the Lachnospiraceae family, 1 ASV of *Eubacterium ruminantium*, *Clostridium butyricum*, *Bacteroides fragilis*, *Succinivibrio*, *Roseburia*, *Bacteroides plebeius*, were less abundant in these patients’ gut than in the gut of patients with spleen qi deficiency and stomach heat. A higher abundance of *Bifidobacterium longum* was significantly correlated with lower baseline levels of INF-α in patients with only spleen qi deficiency (FDR_BH < 0.01, **Figure 2B**). Additionally, the high abundance of other species, such as ASV351-*Bifidobacterium*, ASV32-*Bacteroides coprocola*, and ASV24-*Megamonas*, and the rarity of ASV723-Lachnospiraceae family, ASV96-*Eubacterium ruminantium* group, ASV41-*Bacteroides fragilis*, and ASV76-*Succinivibrio*, showed significant positive correlations with INF-α levels (FDR_BH < 0.05, **Figure 2B**).

### 3.2 Insomnia improvement with herbal formula interventions

**Figure 3:**
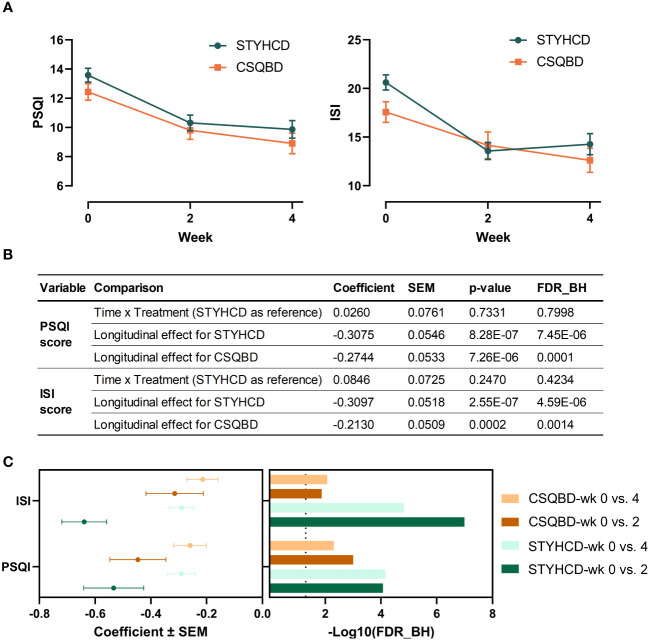
Both herbal formula interventions significantly improved insomnia. **(A)** Changes in PSQI and ISI scores over time following intervention with two herbal formulas. **(B)** Longitudinal effects on PSQI and ISI scores between two treatments and within each treatment. **(C)** Long-term sustainability of the effectiveness of treatments compared to baseline. FDR_BH, FDR-corrected p-value obtained using the Benjamini-Hochberg method to adjust for multiple comparisons. The vertical dashed line represents the FDR_BH threshold of 0.05. P-values were determined using linear mixed models. PSQI, Pittsburgh Sleep Quality Index; ISI, Insomnia Severity Index; STYHCD, spleen-tonifying and yin heat-clearing decoction; CSQBD, center-supplementing and qi-boosting decoction.

Following 4 weeks of treatment, both herbal formulas significantly improved PSQI and ISI scores (**Figures 3A, B**), suggesting consistent longitudinal alleviation of insomnia symptoms. Notably, this improvement was already significant after 2 weeks of treatment and sustained through 4 weeks (**Figure 3C**). However, the effect sizes of both treatments were more substantial between week 2 and 0 than between week 4 and 0. This pattern may stem from treatment compliance and patient adaptation. Besides, no significant differences in insomnia improvement were observed between the two treatments over time (**Figure 3B**).

Apart from insomnia measurements, we evaluated the effects of herbal formula interventions on stress and systematic inflammation. At the baseline, insomnia patients exhibiting only spleen qi deficiency displayed elevated cortisol levels — an indication of heightened stress. This elevation, though initially significant, became statistically insignificant after adjustments for multiple comparisons (**Table 1**). However, CSQBD administration reduced plasma cortisol levels, suggesting its efficacy in mitigating stress in the affected patients. Additionally, after 4-week CSQBD treatment, a marked increase in the levels of anti-inflammatory marker IL-10 along with a reduction in IFN-α levels was observed (**Table S4**). These findings collectively indicate CSQBD’s potential anti-inflammatory and anti-stress effects.

Conversely, patients with combined spleen qi deficiency and stomach heat exhibited significantly higher IFN-α levels than patients with only spleen qi deficiency — a trend that persisted even after FDR correction for multiple tests (**Table 1**). This pattern underscores a more pronounced inflammatory state in these patients. Four-week STYHCD treatment in these patients resulted in a significant increase in the levels of IL-10. Conversely, the levels of inflammatory markers IL-1β and IL-6 and TNF-α also increased significantly (**Table S4**), presenting a complex interplay of inflammatory responses post-treatment of STYHCD.

### 3.3 Impact of herbal formula interventions on gut microbiome

**Figure 4:**
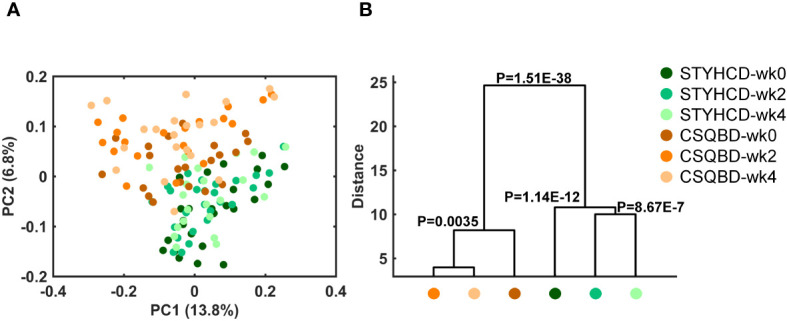
Temporal alterations in the gut microbiome of insomnia patients over time after receiving two herbal formula treatments. **(A)** Principal coordinate analysis based on Unweighted UniFrac distance. **(B)** Clustering of gut microbiota based on inter-group distances obtained through MANOVA test using the initial 31 PCs (explained >80% variation) of unweighted UniFrac PCoA. STYHCD, spleen-tonifying and yin heat-clearing decoction; CSQBD, center-supplementing and qi-boosting decoction.

As indicated by unweighted UniFrac PCoA, both CSQBD and STYHCD treatments significantly changed gut microbiome profiles over the intervention period (**Figure 4**). Similar findings were observed using PCoA of other distance metrics, including Jaccard and Bray-Curtis (**Supplementary Figure S3**). However, no significant longitudinal effects were detected within each treatment group or between the two treatments in terms of alpha-diversity measures, such as Shannon entropy, Pielou’s evenness, and Faith’s phylogenetic diversity (**Supplementary Table S5**).

### 3.4 Key gut microbial species altered by herbal formulas

**Figure 5:**
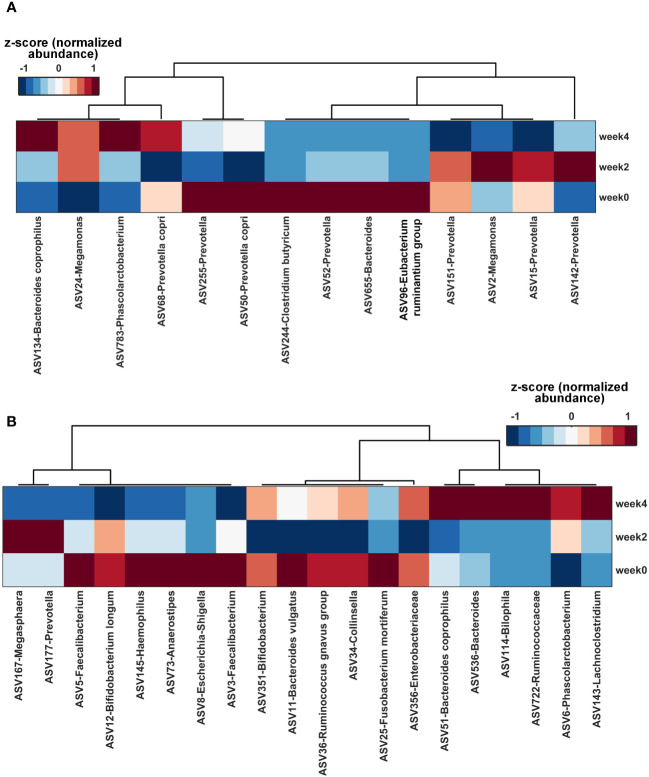
Key gut microbial species altered by herbal formula treatments over time. **(A)** Heatmap of the top 14 gut microbial species altered by STYHCD treatment overtime. **(B)** Heatmap of the top 20 gut microbial species altered by CSQBD treatment overtime. STYHCD, spleen-tonifying and yin heat-clearing decoction; CSQBD, center-supplementing and qi-boosting decoction.

Utilizing a nested stratified 10-fold cross-validated random forest regressor, we identified key gut microbial features impacted by each herbal formula treatment over time. Based on the ranking of the feature importance of these microbes (**Supplementary Figure S4**), top 14 and 20 microbial species were identified for further analysis of STYHCD and CSQBD treatments, respectively. Both treatments enriched *Bacteroides coprophilus* (**Figure 5**).

Among the top 14 microbial species affected by STYHCD (**Figure 5A**), the abundance of 5 species, namely, ASV134-*Bacteroides coprophilus*, ASV783-*Phascolarctobacterium*, ASV244-*Clostridium butyricum*, ASV68-*Prevotella copri*, and ASV24-*Megamonas*, was enhanced. Conversely, the abundance of 9 species, including 5 species of *Prevotella*, ASV244-*Clostridium butyricum*, ASV2-*Megamonas*, ASV96-*Eubacterium ruminantium* group, and ASV655-*Bacteroides*, was inhibited.

Of the top 20 microbial species influenced by CSQBD (**Figure 5B**), 8 species, including ASV51-*Bacteroides coprophilus*, ASV536-*Bacteroides*, ASV177-*Prevotella*, ASV143-*Lachnoclostridium*, ASV114-*Bilophila*, ASV722-Ruminococcaceae family, ASV34-*Collinsella*, and ASV6-*Phascolarctobacterium*, were enriched. In contrast, 12 species, including ASV167-*Megasphaera*, ASV356-Enterobacteriaceae family, ASV3-*Faecalibacterium*, ASV351-*Bifidobacterium*, ASV145-*Haemophilus*, ASV25-*Fusobacterium mortiferum*, ASV11-*Bacteroides vulgatus*, ASV5-*Faecalibacterium*, ASV12-*Bifidobacterium longum*, ASV73-*Anaerostipes*, ASV8-*Escherichia*-*Shigella*, and ASV36-*Ruminococcus gnavus* group, were inhibited.

### 3.5 Longitudinal integrative networks between insomnia improvement, gut microbiome, and systemic inflammation

**Figure 6:**
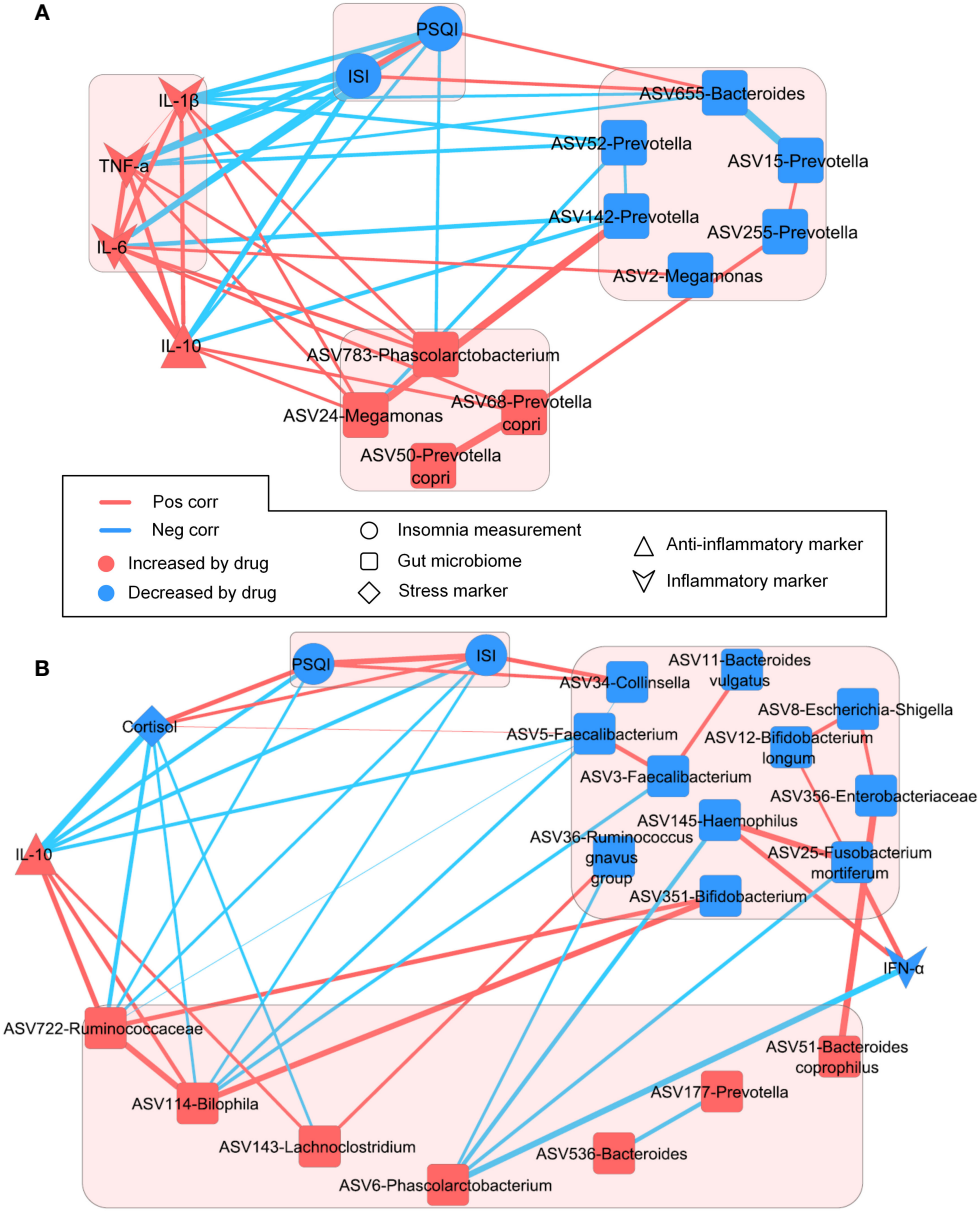
Longitudinal networks involved in insomnia improvement, key gut microbial species, and plasma biomarkers. **(A)** Longitudinal integrative network of STYHCD. **(B)** Longitudinal integrative network of CSQBD. STYHCD, spleen-tonifying and yin heat-clearing decoction; CSQBD, center-supplementing and qi-boosting decoction.

To explore the comprehensive link between insomnia improvement, modulation of key gut microbial species, and systemic inflammation induced by the herbal formula treatments, we performed longitudinal integrative network analysis (**Figure 6**). In the patients with spleen qi deficiency and stomach heat (**Figure 6A**), PSQI and ISI scores were inversely associated with plasma IL-10 levels. ASV783-*Phascolarctobacterium* — enriched by STYHCD treatment — was negatively associated with PSQI and ISI scores, whereas ASV655-*Bacteroides* — inhibited by STYHCD treatment — showed a positive association with PSQI and ISI scores. These findings suggest a link between the change in gut microbiome and improved sleep quality.

In the patients with only spleen qi deficiency (**Figure 6B**), the stress marker cortisol and ASV34-*Collinsella* — both reduced by CSQBD treatment — showed a positive association with PSQI and ISI scores. The anti-inflammatory marker IL-10 along with ASV114-*Bilophila* and ASV722-Ruminococcaceae family showed negative correlations with PSQI and ISI scores. IL-10 was positively associated with both ASV114-*Bilophila* and ASV722-Ruminococcaceae family. This suggests that CSQBD treatment may bolster the immune system — linked to ASV114-*Bilophila* and ASV722-Ruminococcaceae family enrichment in the gut — and improve sleep quality in patients with spleen qi deficiency.

## 4 Discussion

Contrary to the uniform treatment approach of Western medicine, TCM tailors therapies to every patient’s unique TCM syndrome and diagnosis (Huang et al., 2022; Wu et al., 2022). In this study, we found that distinct TCM syndromes in insomnia patients were mirrored in their gut microbiome composition. The significant microbiome variations observed in patients with spleen qi deficiency and those with spleen qi deficiency coupled with stomach heat underscore the intricate connection between gut microbiota and TCM symptomatology. This association was particularly evident from the differential abundance of specific microbial species, such as *Bifidobacterium longum*. This species showed a notable negative correlation with inflammation markers, such as INF-α. Such gut microbiome distinctions in different TCM syndromes were observed in other conditions, such as intestinal diseases and metabolic syndromes (Zhang et al., 2019; Wang et al., 2020; Shang et al., 2022). Our findings further extend the understanding of insomnia, supporting the biological basis of TCM syndrome differentiation. This insight allows precise treatment selection and medication prescriptions, bridging the TCM theory with precision medicine.

Additionally, our study showed that both CSQBD and STYHCD treatments significantly improved the sleep quality — reflected through PSQI and ISI scores. This finding aligns with TCM’s principle of symptom-based treatment and underscores the relevance of personalized approaches in modern medical practice (Janssen et al., 2018; Li et al., 2019). The rapid and sustained improvements in sleep quality highlight the potential efficacy of these herbal formulas. The diminishing effectiveness observed from week 2 to week 4 suggests stabilization in the treatment response — a pattern observed in other herbal intervention studies (Xu et al., 2015; Tong et al., 2018). The lack of significant differences between the two treatments over time suggests a potential universal mechanism in herbal interventions for insomnia, warranting further investigation.

This study contributes to the growing body of evidence linking gut microbiome alterations to sleep improvement, especially within the context of TCM applications in humans. The significant microbiome changes observed in patients post-treatment provide human data supporting the role of the gut-brain axis in sleep regulation (Sen et al., 2021; Bi et al., 2022; Wang et al., 2022). Our longitudinal integrative network suggests a potential link between specific gut microbes, inflammatory responses, and sleep quality improvement. For example, in insomnia patients with spleen qi deficiency and stomach heat, STYHCD enriched ASV783-*Phascolarctobacterium*, which has been shown to be reduced in patients with obstructive sleep apnea (Szabo et al., 2022). Similarly, CSQBD treatment in the patients exhibiting spleen qi deficiency enriched species, such as ASV114-*Bilophila* and ASV722-Ruminococcaceae, associated with stress and insomnia improvements. The reduction in cortisol levels following CSQBD treatment underscores its potential in stress management — a key factor in insomnia (Zhao et al., 2021; Dressle et al., 2022). Based on our findings, it might be promising to consider the potential beneficial effects of *Phascolarctobacterium* and Ruminococcaceae in the context of insomnia treatment. These bacteria are known as short-chain fatty acids (SCFAs) producers (Wu et al., 2017; Xie et al., 2022). SCFAs, particularly propionate, may influence the gut-brain axis by affecting inflammatory responses, neurotransmitter synthesis, and perhaps even the regulation of stress and circadian rhythms — factors closely linked to the pathophysiology of insomnia (Tahara et al., 2018; Kimura et al., 2020; Cook et al., 2021; Grüter et al., 2023). These findings collectively reinforce the role of TCM in utilizing gut microbiota modulation as a therapeutic pathway for insomnia.

While our study did not directly establish a causative role of the gut microbiome in the effects of TCM formulas on insomnia, it aligns with emerging research suggesting the microbiome’s influence on sleep regulation. Notably, a previous study found that depletion of the gut microbiota by antibiotics significantly affects sleep/wake behavior, potentially through disruptions in neurotransmitter balances, such as serotonin, underscoring the microbiome’s regulatory capacity on sleep (Ogawa et al., 2020). Additionally, a more recent study proposes a causal link between specific gut microbiotas and insomnia via a Mendelian randomized two-way validation method (Wang et al., 2024). These findings highlight the complexity of the gut-brain axis and its implications for sleep disorders. Given the preliminary nature of these insights and the absence of direct evidence from our study, further investigation into how TCM formulas interact with the gut microbiome to influence sleep is crucial. This includes the need for both animal model studies and clinical trials to elucidate the underlying mechanisms more clearly.

While our study provides valuable insights into precision medicine for insomnia patients with distinct TCM syndromes, it is important to acknowledge its limitations. Future research involving larger clinical trials that include healthy subjects, as well as placebo and positive drug control groups, would facilitate a more comprehensive evaluation of TCM’s efficacy in treating insomnia and its link with gut microbiome. Additionally, investigating the molecular mechanisms behind the observed shifts in gut microbiota and sleep quality could unearth deeper insights into how Chinese herbal formulas exert their therapeutic effects on insomnia. Such studies are vital for the seamless integration of traditional herbal formula treatments into modern clinical practices, enhancing the precision of insomnia therapy.

## 5 Conclusion

This study reveals distinct microbiome profiles associated with different TCM syndromes and underscores the link between the gut microbiome and efficacy of Chinese herbal formulas in improving insomnia. These findings not only enrich our understanding of the gut-brain axis in insomnia but also open new avenues for personalized and holistic insomnia treatments using herbal formulas.

## Data availability statement

The sequencing data has been archived to NCBI Sequence Read Archive (BioProject: PRJNA1099612).

## Ethics statement

The studies involving humans were approved by Ethnics Committee of Guangdong Provincial Hospital of Chinese Medicine. The studies were conducted in accordance with the local legislation and institutional requirements. The participants provided their written informed consent to participate in this study.

## Author contributions

HZ: Formal analysis, Investigation, Methodology, Writing – original draft. JX: Data curation, Formal analysis, Visualization, Writing – original draft, Writing – review & editing. LZ: Supervision, Writing – review & editing. ZZ: Supervision, Writing – review & editing. ZF: Formal analysis, Visualization, Writing – review & editing. YXL: Formal analysis, Visualization, Writing – review & editing. CZ: Formal analysis, Investigation, Writing – review & editing. RX: Formal analysis, Investigation, Writing – review & editing. ZFZ: Investigation, Writing – review & editing. YL: Funding acquisition, Project administration, Writing – review & editing. LY: Funding acquisition, Project administration, Writing – review & editing.
