# Supplementary figures and images for: Traditional Chinese herbal formulas modulate gut microbiome and improve insomnia in patients with distinct syndrome types: insights from an interventional clinical study
Source: Front Cell Infect Microbiol. 2024 May 16;14:1395267. doi: 10.3389/fcimb.2024.1395267 (PMC11137223; doi:10.3389/fcimb.2024.1395267)

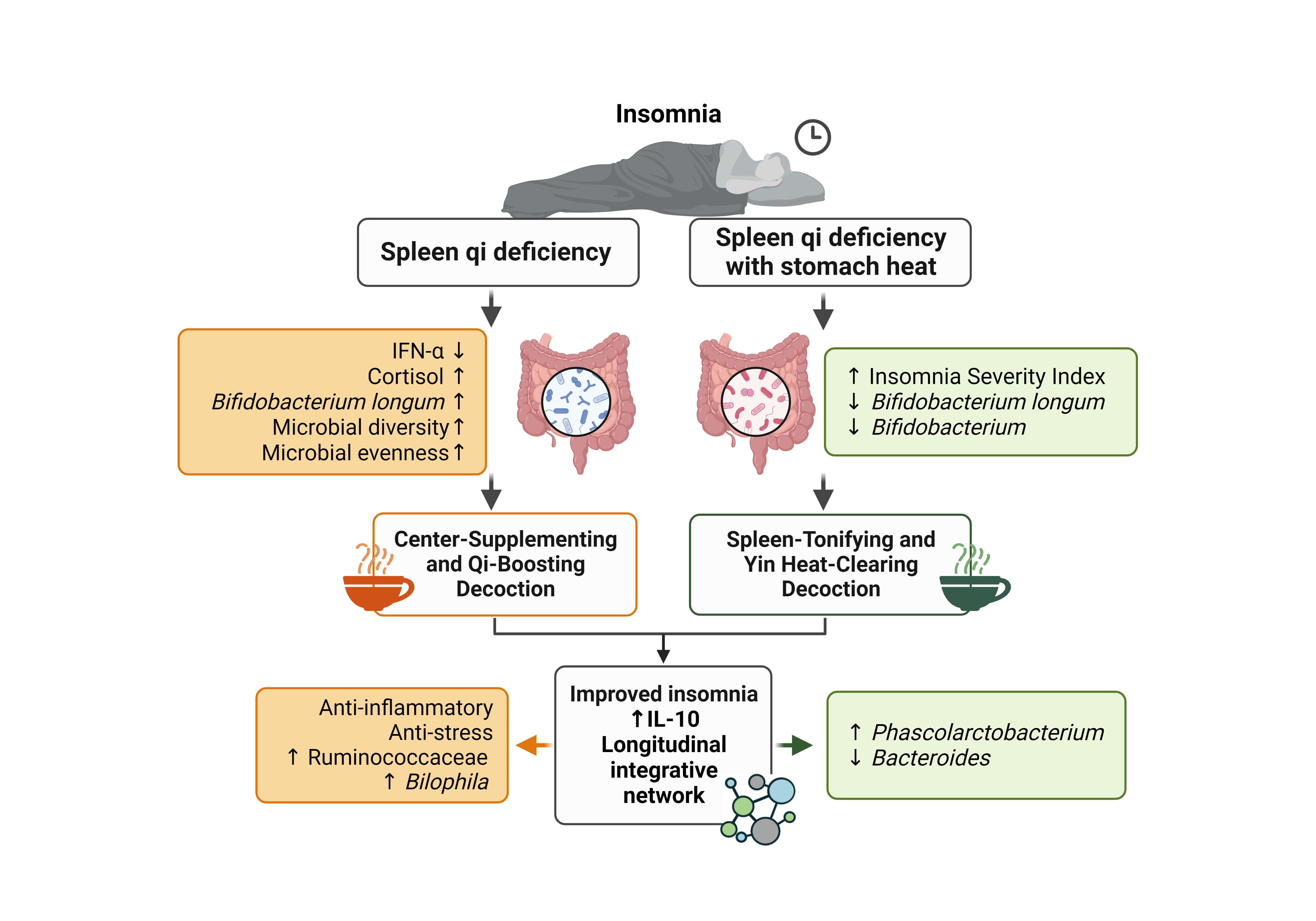

Supplement: Supplementary file 1 [file Image_1.png]
