# Supplementary material for: Traditional Chinese herbal formulas modulate gut microbiome and improve insomnia in patients with distinct syndrome types: insights from an interventional clinical study
Source: Front Cell Infect Microbiol. 2024 May 16;14:1395267. doi: 10.3389/fcimb.2024.1395267 (PMC11137223; doi:10.3389/fcimb.2024.1395267)
Supplement: Supplementary file 2 [file DataSheet_1.pdf]

Supplementary Material

1 Supplementary methods

Standard production process of the decocting-free granules of two herbal formulas

1.1 The STYHCD herbal formula

The STYHCD herbal formula is composed of ten different herbs, as detailed in Supplementary Table S1. The primary components of each herb were extracted following the specified processing procedures outlined below. Subsequently, these ten extracts were thoroughly mixed for 30 minutes to achieve uniformity. The homogenized mixture was then granulated to produce granules with a size range of 12-40 mesh. Finally, the granules were packaged into four aluminum foil bags.

1.1.1 Bupleurum chinense DC.

Decoct the pieces with water twice, first for 2 hours and second for 1 hour. 2. Combine the decoctions, filter, and concentrate the filtrate to a clear paste with a relative density of 1.10 to 1.12 (65±5°C). 3. Spray-dry, sift, and mix.

1.1.2 Glycyrrhiza uralensis Fisch. ex DC.

Decoct the pieces with water twice, first for 2.5 hours and second for 1.5 hour. 2. Combine the decoctions, filter, and concentrate the filtrate to a clear paste with a relative density of 1.09 to 1.11 (65±5°C). 3. Spray-dry, sift, and mix.

1.1.3 Astragalus mongholicus Bunge

Decoct the pieces with water twice, first for 2 hours and second for 1 hour. 2. Combine the decoctions, filter, and concentrate the filtrate to a clear paste with a relative density of 1.11 to 1.13 (65±5°C). 3. Spray-dry, sift, and mix.

1.1.4 Atractylodes macrocephala Koidz.

Decoct the pieces with water twice, first for 2 hours and second for 1 hour. 2. Combine the decoctions, filter, and concentrate the filtrate to a clear paste with a relative density of 1.09 to 1.11 (65±5°C). 3. Spray-dry, sift, and mix.

1.1.5 Hansenia weberbaueriana (Fedde ex H.Wolff) Pimenov & Kljuykov

Decoct the pieces with water twice, first for 2 hours and second for 1 hour. 2. Combine the decoctions, filter, and concentrate the filtrate to a clear paste with a relative density of 1.10 to 1.12 (65±5°C). 3. Spray-dry, sift, and mix

1.1.6 Actaea heracleifolia (Kom.) J.Compton

Decoct the pieces with water for 2.5 hours. 2. Filter and concentrate the filtrate to a clear paste with a relative density of 1.08 to 1.10 (65±5°C). 3. Spray-dry, sift, and mix

1.1.7 Codonopsis pilosula (Franch.) Nannf.

Decoct the pieces with water twice, first for 1.5 hours and second for 0.5 hour. 2. Combine the decoctions, filter, and concentrate the filtrate to a clear paste with a relative density of 1.03 to 1.05 (65±5°C). 3. Spray-dry, sift, and mix

1.1.8 Scutellaria baicalensis Georgi

Decoct the pieces with water twice, first for 1 hour and second for 1 hour. 2. Combine the decoctions, filter, and concentrate the filtrate to a clear paste with a relative density of 1.15 to 1.17 (65±5°C). 3. Spray-dry, sift, and mix

1.1.9 Coptis chinensis Franch.

Decoct the pieces with water twice, first for 1 hour and second for 1 hour. 2. Combine the decoctions, filter, and concentrate the filtrate to a clear paste with a relative density of 1.09 to 1.11 (65±5°C). 3. Spray-dry, sift, and mix

1.1.10 Pothos chinensis (Raf.) Merr.

Decoct the pieces with water for 3 hours. 2. Filter and concentrate the filtrate to a clear paste with a relative density of 1.10 to 1.11 (65±5°C). 3. Spray-dry, sift, and mix

1.2 The CSQBD herbal formula

The CSQBD herbal formula is composed of 8 different herbs, as detailed in Supplementary Table S2. The primary components of each herb were extracted following the specified processing procedures outlined below. Subsequently, these ten extracts were thoroughly mixed for 30 minutes to achieve uniformity. The homogenized mixture was then granulated to produce granules with a size range of 12-40 mesh. Finally, the granules were packaged into four aluminum foil bags.

1.2.1 Astragalus mongholicus Bunge

Decoct the pieces with water twice, first for 2 hours and second for 1 hour. 2. Combine the decoctions, filter, and concentrate the filtrate to a clear paste with a relative density of 1.11 to 1.13 (65±5°C). 3. Spray-dry, sift, and mix.

1.2.2 Glycyrrhiza uralensis Fisch. ex DC.

Decoct the pieces with water twice, first for 2.5 hours and second for 1.5 hour. 2. Combine the decoctions, filter, and concentrate the filtrate to a clear paste with a relative density of 1.09 to 1.11 (65±5°C). 3. Spray-dry, sift, and mix.

1.2.3 Codonopsis pilosula (Franch.) Nannf.

Decoct the pieces with water twice, first for 1.5 hours and second for 0.5 hour. 2. Combine the decoctions, filter, and concentrate the filtrate to a clear paste with a relative density of 1.03 to 1.05 (65±5°C). 3. Spray-dry, sift, and mix.

1.2.4 Angelica sinensis (Oliv.) Diels

Decoct the pieces with water for 2.5 hours. 2. Filter and concentrate the filtrate to a clear paste with a relative density of 1.07 to 1.09 (65±5°C). 3. Spray-dry, sift, and mix.

1.2.5 Citrus reticulata Blanco

Decoct the pieces with water for 1 hours. 2. Filter and concentrate the filtrate to a clear paste with a relative density of 1.05 to 1.07 (65±5°C). 3. Spray-dry, sift, and mix.

1.2.6 Actaea heracleifolia (Kom.) J.Compton

Decoct the pieces with water for 2.5 hours. 2. Filter and concentrate the filtrate to a clear paste with a relative density of 1.08 to 1.10 (65±5°C). 3. Spray-dry, sift, and mix.

1.2.7 Bupleurum chinense DC.

Decoct the pieces with water twice, first for 2 hours and second for 1 hour. 2. Combine the decoctions, filter, and concentrate the filtrate to a clear paste with a relative density of 1.10 to 1.12 (65±5°C). 3. Spray-dry, sift, and mix.

1.2.8 Atractylodes macrocephala Koidz.

Decoct the pieces with water twice, first for 1.5 hours and second for 1 hour. 2. Combine the decoctions, filter, and concentrate the filtrate to a clear paste with a relative density of 1.05 to 1.07 (65±5°C). 3. Spray-dry, sift, and mix.

1.3 Detection of the main chemicals in the two herbal formulas

The samples were dissolved in hot water and then extracted once with an equal volume of butyl alcohol. The butyl alcohol fraction was concentrated until dry, and subsequently dissolved in methanol. This methanol solution was then filtered through 0.22 μm micropore filter for further analysis.

High Performance Liquid chromatography (HPLC) was performed using an Accela system (Thermo Scientific, USA). The chromatographic separation was conducted on an Agilent Eclipse Plus C18 column (4.6 mm × 100 mm, 2.7 μm). The mobile phase consisted of 0.05% formic acid solution (A) and acetonitrile (B). The gradient elution program was set as follows: 0 min, A:B 90:10; 15 min, A:B 75:25; 40 min, A:B 60:40; 55 min, A:B 30:70; 56 min, A:B 90:10; 60 min, A:B 95:5. The flow rate was 0.3 mL/min and the injection volume was 2 μL.

LC-MS analysis was performed on LTQ-Orbitrap XL Mass Spectrometer (Thermo Scientific, USA) equipped with an ESI source in negative ionization mode. The capillary temperature was set at 350℃. Sheath, auxiliary, and sweep gas flow rates were set at 30, 4, and 0 units, respectively. Source voltage was set at 3.8 kV, capillary voltage at -18 V, and tube lens voltage at -67 V. The data evaluation was performed with Xcalibur 2.0 software. A Collision-Induced Dissociation (CID) energy of 35 eV was used in a data dependent scan mode in MS/MS analysis.

2 Supplementary Figures and Supplementary Tables

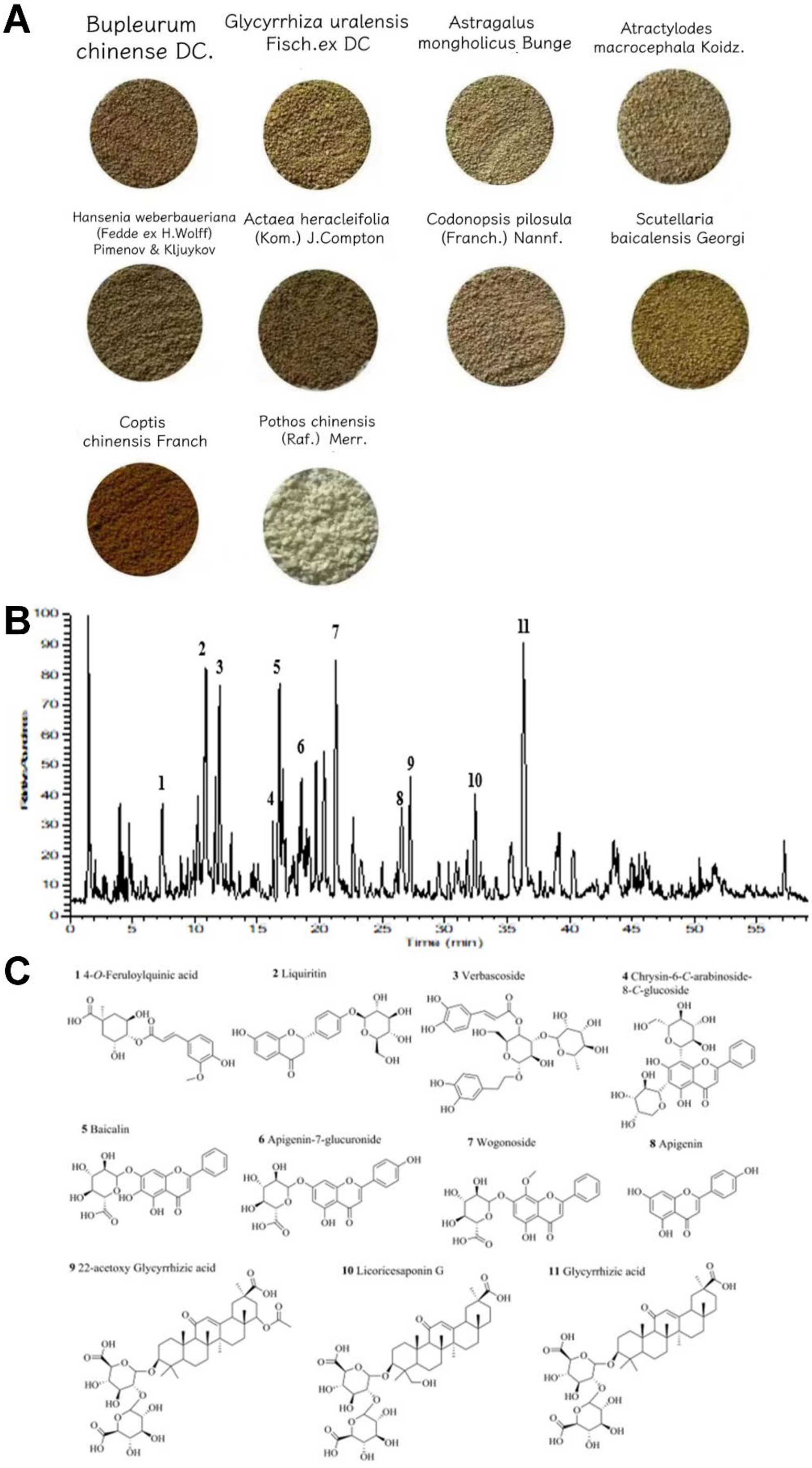

Supplementary Figure S1. The STYHCD herbal formula. (a) Photos of the decocting-free granules of the ten herbs in the STYHCD herbal formula. (b) HPLC-TIC-MS finger-printing of STYHCD herbal formula. (c) The eleven main chemicals in STYHCD.

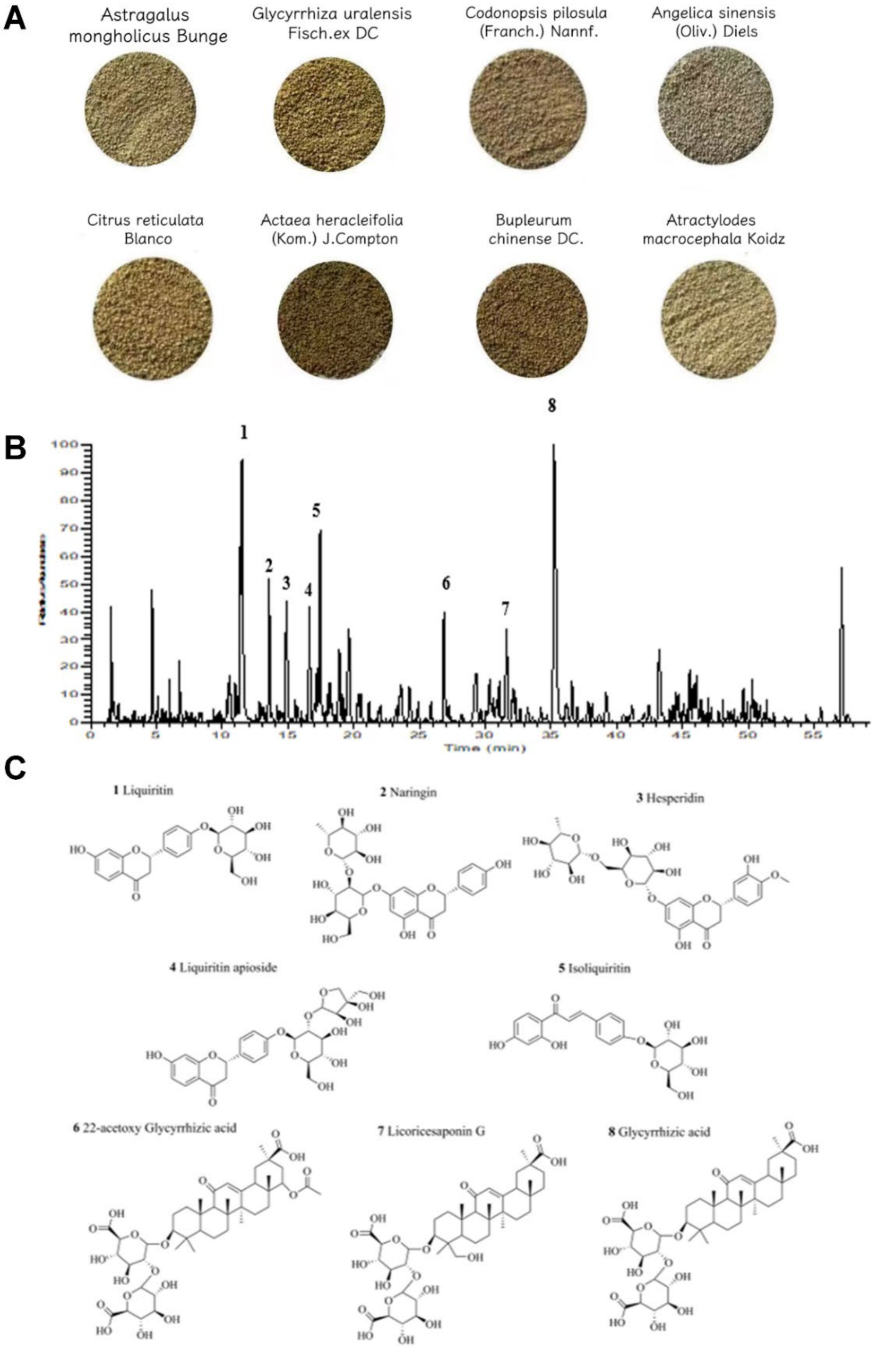

Supplementary Figure S2. The CSQBD herbal formula. (a) Photos of the decocting-free granules of the eight herbs in the CSQBD herbal formula. (b) HPLC-TIC-MS finger-printing of CSQBD herbal formula. (c) The eight main chemicals in CSQBD. STYHCD, spleen-tonifying and yin heat-clearing decoction; CSQBD, center-supplementing and qi-boosting decoction.

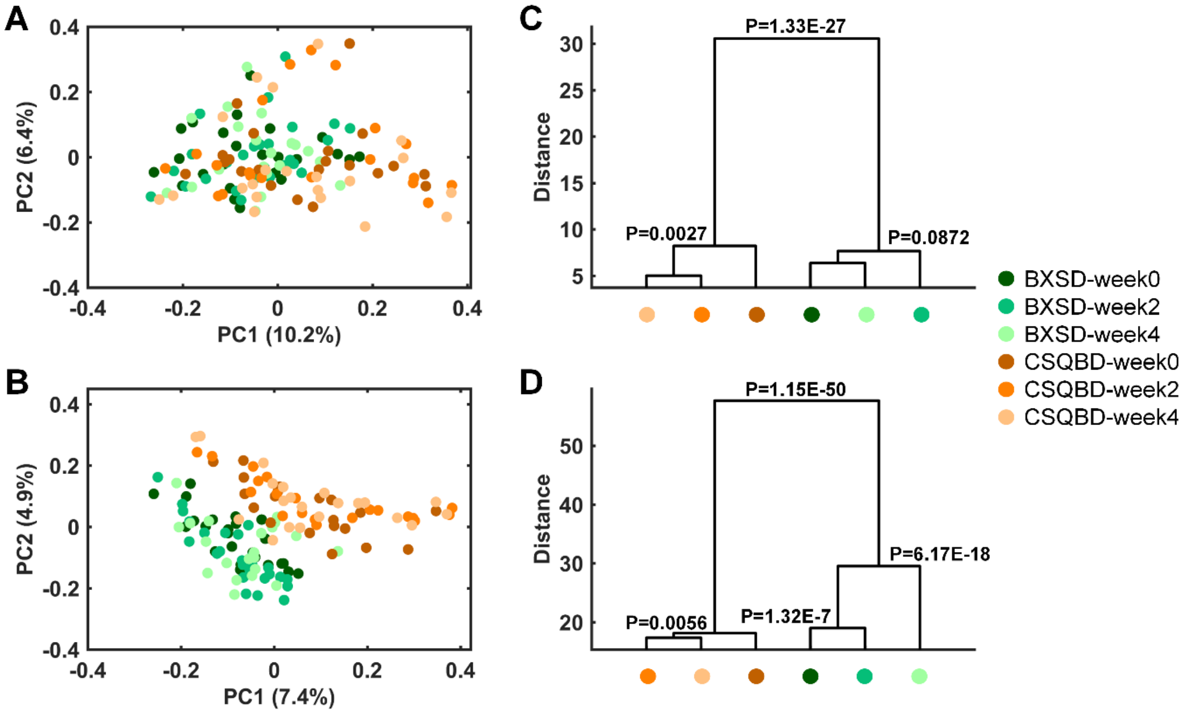

Supplementary Figure S3. The temporal alteration of the gut microbiome in insomnia patients received two different herbal formula treatments over time. Principal coordinate analysis based on (A) Bray-Curtis dissimilarity distance and (B) Jaccard distance. Clustering of gut microbiota based on inter-group distances obtained through MANOVA test based on (C) Bray-Curtis dissimilarity distance and (D) Jaccard distance.

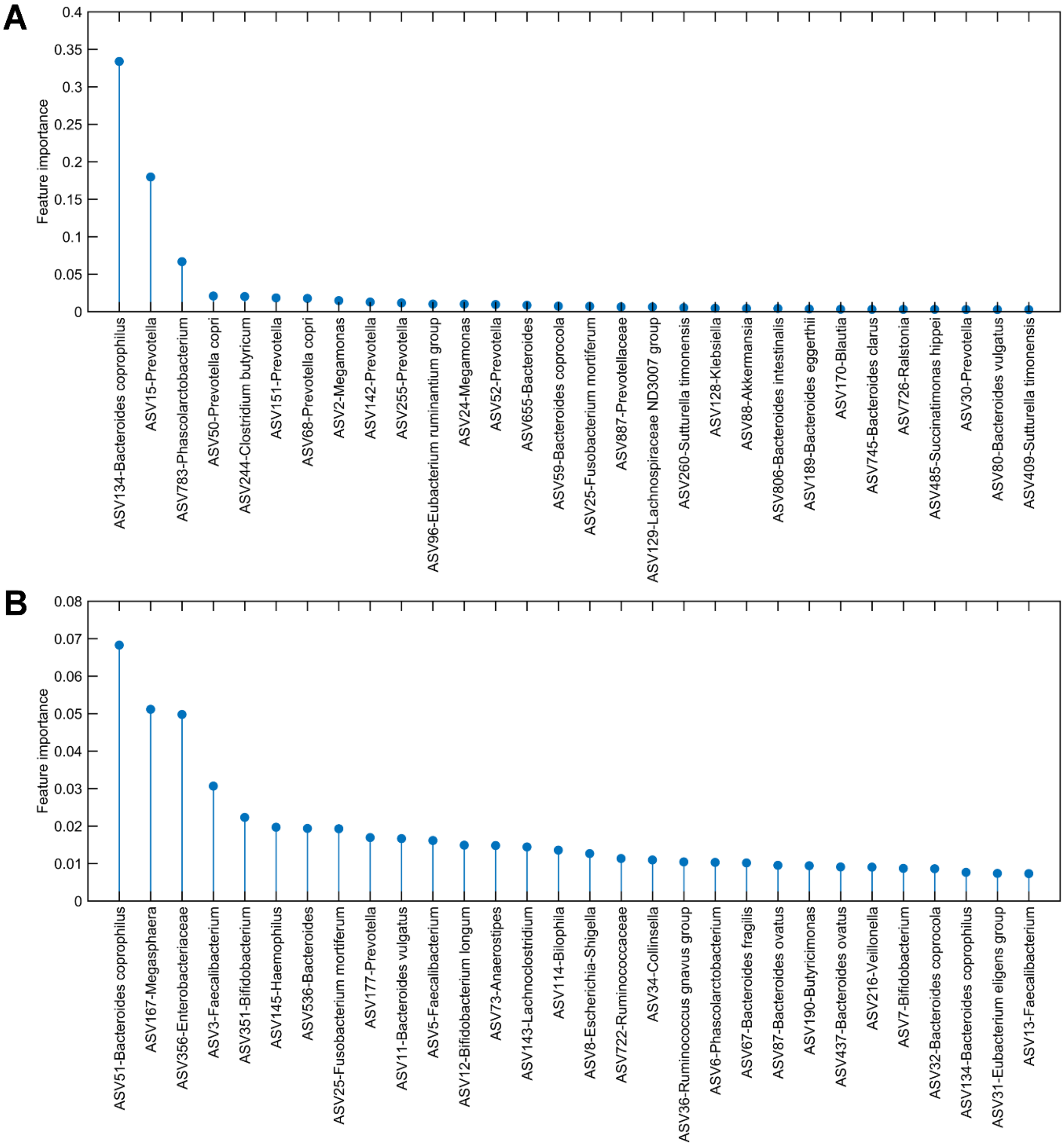

Supplementary Figure S4. Feature importance of the top 30 gut microbial species identified through nested 10-fold cross-validated random forest regression for each herbal formula. A. STYHCD, Spleen-Tonifying and Yin Heat-Clearing Decoction. B. CSQBD, Center-Supplementing, and Qi-Boosting Decoction.

Supplementary Table S1. Herbal ingredients of the Spleen-Tonifying and Yin Heat-Clearing Decoction.

Herb	Weight of the herb (g)	Herbal decocting-free granule (g)
Bupleurum Chinese DC.	15	3.9
Glycyrrhiza uralensis Fisch. ex DC.	10	4.3
Astragalus mongholicus Bunge	10	3.7
Atractylodes macrocephala Koidz.	10	5.8
Hansenia weberbaueriana (Fedde ex H.Wolff) Pimenov & Kljuykov	10	2.5
Actaea heracleifolia (Kom.) J.Compton	5	0.7
Codonopsis pilosula (Franch.) Nannf.	5	4.9
Scutellaria baicalensis Georgi	5	1.9
Coptis chinensis Franch.	5	0.9
Gypsum ribrosum	5	1
Total weight	80	29.6

Supplementary Table S2. Herbal ingredients of the Center-Supplementing, and Qi-Boosting Decoction.

Herb	Weight of the herb (g)	Herbal decocting-free granule (g)
Astragalus mongholicus Bunge	30	10
Glycyrrhiza uralensis Fisch. ex DC	15	5.8
Atractylodes macrocephala Koidz.	15	9.5
Codonopsis pilosula (Franch.) Nannf.	10	8.9
Angelica sinensis (Oliv.) Diels	10	6.4
Citrus reticulata Blanco	10	3.7
Actaea heracleifolia (Kom.) J.Compton	10	1.3
Bupleurum chinense DC.	10	2.3
Total weight	110	47.9

Supplementary Table S3. Baseline alpha-diversity for the insomnia patients with different TCM symptoms.

alpha-diversity metric	Coefficient	SEM	p-value	FDR_BH
shannon_entropy	0.0011	0.0004	0.0072	0.0249
pielou_evenness	0.0002	6.76E-05	0.0083	0.0249
chao1	-0.0006	0.0013	0.6654	0.9489
observed_features	-0.0003	0.0012	0.7907	0.9489
menhinick	1.41E-05	4.20E-05	0.7390	0.9489
margalef	-7.86E-06	0.0002	0.9662	0.9662

STYHCD group was used as the reference.

FDR_BH, FDR-corrected p-value were obtained with the Benjamini-Hochberg method to adjust for multiple comparisons.

Supplementary Table S4. Longitudinal effects of the two herbal formulas on stress and systematic inflammation over time.

Treatment	Cytokine	Coefficient	SEM	p-value	FDR_BH
STYHCD	Cortisol (ng/mL)	0.0194	0.0477	0.6876	0.8251
	IL-1β (pg/mL)	0.1909	0.0642	0.0064	0.0166
	IL-6 (pg/mL)	0.1435	0.0364	0.0008	0.0037
	TNF-α (pg/mL)	0.2013	0.0615	0.0033	0.0098
	IFN-α (pg/mL)	-0.0846	0.0721	0.2471	0.3706
	IL-10 (pg/mL)	0.1291	0.0456	0.0102	0.0229
	TNF-β (pg/mL)	0.0186	0.0741	0.8026	0.8498
CSQBD	Cortisol (ng/mL)	-0.1372	0.0760	0.0788	0.1772
	IL-1β (pg/mL)	-0.0419	0.0779	0.5965	0.6316
	IL-6 (pg/mL)	0.1026	0.0725	0.1730	0.3115
	TNF-α (pg/mL)	0.0688	0.0794	0.3914	0.5419
	IFN-α (pg/mL)	-0.1606	0.0748	0.0381	0.1142
	IL-10 (pg/mL)	0.2042	0.0730	0.0081	0.0362
	TNF-β (pg/mL)	-0.0655	0.0816	0.4278	0.5500

FDR_BH, FDR-corrected p-value were obtained with the Benjamini-Hochberg method to adjust for multiple comparisons.

STYHCD, spleen-tonifying and yin heat-clearing decoction; CSQBD, center-supplementing and qi-boosting decoction.

Supplementary Table S5. Longitudinal effects of the two herbal formulas on the alpha-diversity of the gut microbiome over time.

Treatment	alpha-diversity metric	Coefficient	SEM	p-value	FDR_BH
STYHCD	shannon_entropy	-1.64E-04	1.35E-04	0.2270	0.5537
	pielou_evenness	-3.12E-05	2.01E-05	0.1247	0.5537
	chao1	6.31E-04	6.54E-04	0.3374	0.5537
	observed_features	-5.51E-04	6.09E-04	0.3691	0.5537
	menhinick	2.09E-05	3.16E-05	0.5104	0.6125
	margalef	1.43E-05	1.20E-04	0.9055	0.9055
CSQBD	shannon_entropy	3.85E-04	3.00E-04	0.2040	0.6120
	pielou_evenness	6.77E-05	5.09E-05	0.1887	0.6120
	chao1	-1.17E-04	5.45E-04	0.8302	0.8302
	observed_features	-1.52E-04	5.02E-04	0.7623	0.8302
	margalef	-4.56E-05	8.63E-05	0.5994	0.8302
	menhinick	-4.52E-06	2.04E-05	0.8257	0.8302

FDR_BH, FDR-corrected p-value were obtained with the Benjamini-Hochberg method to adjust for multiple comparisons.

STYHCD, spleen-tonifying and yin heat-clearing decoction; CSQBD, center-supplementing and qi-boosting decoction.
